# Are health workers reduced to being drug dispensers of antiretroviral treatment? A randomized cross-sectional assessment of the quality of health care for HIV patients in northern Uganda

**DOI:** 10.1093/heapol/czz074

**Published:** 2019-08-13

**Authors:** Ulrike G Seeberger, Joseph J Valadez

**Affiliations:** Department of International Health, Liverpool School of Tropical Medicine, Pembroke Place, Liverpool, UK

**Keywords:** Antiretroviral therapy, quality of care, HIV/AIDS treatment, Uganda, direct observation

## Abstract

High quality of care (QoC) for antiretroviral treatment (ART) is essential to prevent treatment failure. Uganda, as many sub-Saharan African countries, increased access to ART by decentralizing provision to districts. However, little is known whether this rapid scale-up maintained high-quality clinical services. We assess the quality of ART in the Acholi and Lango sub-regions of northern Uganda to identify whether the technical quality of critical ART sub-system needs improvement. We conducted a randomized cross-sectional survey among health facilities (HF) in Acholi (*n* = 11) and Lango (*n* = 10). Applying lot quality assurance sampling principles with a rapid health facility assessment tool, we assessed ART services vis-à-vis national treatment guidelines using 37 indicators. We interviewed health workers (*n* = 21) using structured questionnaires, directly observed clinical consultations (*n* = 126) and assessed HF infrastructure, human resources, medical supplies and patient records in each health facility (*n* = 21). The district QoC performance standard was 80% of HF had to comply with each guideline. Neither sub-region complied with treatment guidelines. No HF displayed adequate: patient monitoring, physical examination, training, supervision and regular monitoring of patients’ immunology. The full range of first and second line antiretroviral (ARV) medication was not available in Acholi while Lango had sufficient stocks. Clinicians dispensed available ARVs without benefit of physical examination or immunological monitoring. Patients reported compliance with drug use (>80%). Patients’ knowledge of preventing HIV/AIDS transmission concentrated on condom use; otherwise it was poor. The poor ART QoC in northern Uganda raises major questions about ART quality although ARVs were dispensed. Poor clinical care renders patients’ reports of treatment compliance as insufficient evidence that it takes place. Further studies need to test patients’ immunological status and QoC in more regions of Uganda and elsewhere in sub-Saharan Africa to identify topical and geographical areas which are priorities for improving HIV care.

**Key Messages**

- Uganda’s low-quality clinical care for antiretroviral therapy raises worries about it compromising patients’ immunological status and drug resistance developing.
- Policies aimed at scaling up effective therapies need to be accompanied by keen assessment and improvement measures of the quality of clinical care.
- Direct observation of health services produced greater insight about the quality of care than patient records and service availability reports.

## Introduction

Uninterrupted access to treatment with high-quality clinical care is essential for people living with HIV/AIDS (PLHA) to achieve good health outcomes. On the one hand, a patient’s perceived quality of care (QoC) (compassion, confidentiality, timeliness, communication and information) is associated with adherence to treatment (Etienne *et al.*, 2010) and hence, with avoidance of therapy failure and drug resistance (Osterberg and Blaschke, 2005). On the other hand, regular clinical and immunological assessment and taking relevant treatment consequences is also crucial for reducing risk factors for poor clinical outcomes. Accordingly, improved QoC for PLHA can reduce related morbidity and mortality (Ahoua *et al.*, 2009; Lawn *et al.*, 2010). Depending on the intensity of monitoring efforts and the application of different lines of treatment approximately 2.4–3.5 million disability adjusted life years (DALYs) could be averted yearly by antiretroviral treatment (ART) in sub-Saharan Africa (Hogan *et al.*, 2005).

By 2014, Uganda achieved approximately 50% coverage with treatment of eligible PLHA (World Health Organisation, 2003; UNAIDS, 2014). Alongside the new eligibility criteria introduced in 2013/14 (Uganda Ministry of Health, 2013), including more patients in the therapy scheme, the coverage is expected to have dropped again afterwards. ART in sub-Saharan Africa demands between $556 and $2010 per DALY averted depending on the effort in clinical monitoring of patients and expenditure in different types of antiretroviral medication (ARV) (Hogan *et al.*, 2005; Mikkelsen *et al.*, 2017). The national health budget could only cover 12% of Uganda’s expenses for HIV/AIDS programmes in 2012/13 (UNAIDS, 2015). Furthermore, ART care is supported by an estimated one-third of all health system human resources (HR) (USAID, 2010). However, HR in Uganda’s health system are scarce; in 2013, 37% of posts were vacant (Ministry of Health, 2013); in northern Uganda, 49% were vacant during 2012 (NUMAT, 2012). This raises questions about whether quality of ART care (QoC) can be obtained due to the immense caseload in addition to the HR crisis and the enormous funding gap.

Although Uganda’s Ministry of Health prioritizes the strengthening of monitoring and evaluation of QoC for HIV/AIDS services (Uganda AIDS Commission, 2011), few institutions measure quality beyond service availability and outcome indicators (NUMAT, 2010, 2011, 2012; Ministry of Health, 2011b; Ministry of Health, 2012a,b). Data on relevant specific clinical practices influencing patients’ outcomes are not available. Even international indicators for quality ART care in low- and middle-income countries do not include performance variables even though acknowledging the importance of on-site QoC (Ahonkhai *et al.*, 2012).

Our study seeks to address this information gap for patients on ART through a QoC assessment in northern Uganda, which has a serious scarcity of health workers (HW), and hence, is a location where deficiencies in quality services might be more apparent than elsewhere. The question we address is: what is the current QoC received by ART patients when assessed using multiple standards of care as stipulated in the national guidelines? The different technical categories of the quality assessed allow to prioritize clinical and geographic areas that score worse. The resulting information can lead programme managers to invest resources in targeted strategies. This result should lead to improved diagnostic and therapeutic procedures, counselling and treatment outcomes, which potentially include a reduced viral load and drug resistance among PLHA. This innovative approach can be applied elsewhere in sub-Saharan Africa.

## Methods

The sampling frame for this study comprises ART health facilities (HF) in two sub-regions of northern Uganda. During 2014, 80 HF provided ART services in the Acholi sub-region; 64 in Lango. To assess routine ART provision, only facilities attending at least 12 non-naive ART patients per week were included in the sampling frame (Acholi: *N* = 20; Lango: *N* = 21) (Ministry of Health, 2014). We stipulated these inclusion criteria to permit the data collection to take place in 1 day and to prioritize facilities that see the largest number of patients. This study therefore concerns HFs who regularly serve PLHA.

The Rapid Health Facility Assessment (R-HFA) tool we pretested in eastern Uganda in 2011 (Deurman, 2011), and refined in 2014 is attached as Supplementary data. The instrument comprised five modules: direct observation of the clinical consultation; patient interview; clinical documentation; checklist of drugs, laboratory and other equipment; and HW interview. Data from those modules measured 37 indicators/guidelines. The structure and protocol for this R-HFA were adapted from a version developed for assessing clinical services for sick children (Oladele *et al.*, 2012; Berendes *et al.*, 2014) to which a co-author contributed (www.mchip.net/technical-resource/the-rapid-health-facility-assessment-r-hfa/).

We applied lot quality assurance sampling (LQAS) principles to calculate sub-regional random sample sizes. LQAS does not calculate a precise prevalence or coverage measure of QoC or other attributes (Robertson *et al.*, 1997b) but uses binomial probabilities to determine the probability a target prevalence for a certain characteristic in a defined population has not been reached. For this purpose, LQAS is quite robust (Pagano and Valadez, 2010). With this information, policy and decision-makers identify priority geographical and technical areas for corrective action and to apportion resources accordingly (Robertson *et al.*, 1997a; Brooker *et al.*, 2005).

We also adapted the protocol used successfully for assessing the QoC of maternal and child health services (Oladele *et al.*, 2012; Berendes *et al.*, 2014) to this assessment of ART services, and continued to use as the standard that 80% of facilities of one sub-region needed to comply with the national guidelines for ART services for a sub-region to be classified as reaching an acceptable QoC. In LQAS parlance, this target is referred to as an upper threshold (p_U_). The 80% target was measured separately for each of the 37 indicators. Simultaneously, we set a lower threshold (p_L_) to identify sub-regions with very low QoC for these indicators. Typically, p_L_ is 30 percentage points below p_U_ (p_L_ = 50%).

The binomial model is often used to calculate LQAS cut-off values and errors to assess coverage in populations. However, HF assessments typically have sampling frames with smaller numbers of elements and require a finite population correction to calculate the cut-off values: we used the above LQAS parameters in the hypergeometric model (Valadez, 1991) to calculate HF sample sizes and cut-off values (*d*) to classify the sub-regions such that α and β errors (the risk of wrongly classifying a sub-region as not reaching the quality threshold and vice versa) did not exceed 0.10. Cut-off values are the minimal number of individuals in the sample that must have the trait of interest for the lot (or sub-region) to be accepted. Otherwise it is classified as failing to reach the target. Sub-regions with performance between p_U_ and p_L_ had a probability of classification associated with its proximity to either threshold. The resulting sample sizes, cut-off values and error terms were: Acholi: *n* = 11, cut-off = 8, α = 0.055, β = 0.064; Lango: *n* = 10, cut-off = 7, α  = 0.049, β  = 0.095 (Table 1). Each indicator we measured separately and applied LQAS principles to assess its performance in the sub-region using these parameters.

**Table 1:** Sample size, decision rule and classification errors for Acholi, Lango and northern Uganda

Sub-region	No. eligible HF (No.)^a^	p_U_	p_L_	Sample Size (*n*)	Decision Rule (*d*)	α error	β error
Acholi (original sample)	20	0.8	0.5	10	7	0.043	0.089
Acholi (additional facility added)^a^	20	0.8	0.5	11	8	0.068	0.035
Lango	21	0.8	0.5	10	7	0.049	0.095

a In Acholi, we included one additional HF as a contingency in case of locating an inaccessible HF the resulting data were included in the analysis (see Acholi—additional facility added).

HF, health facilities.

For a health facility to meet the required standard of quality for clinical performance, clinical behaviour had to be consistent with the national guidelines 95% of the time (p_u_ = 0.95). We assumed that clinical performance is bimodally distributed: clinicians know the proper technique and use it, or they are ignorant of it and therefore do not. For this assessment, we therefore use a wider range to separate p_U_ and p_L_. The resulting parameters we used were p_U_ = 95%, p_L_ = 50%, *n* = 6, cut-off = 5, α = 0.033 and β = 0.109. Therefore, clinicians had to act correctly for each guideline in five out of six cases for the HF to be classified as having an acceptable QoC (Valadez, 1991).

For the observation of clinical performance, we sampled the most experienced clinician on the day of the visit to observe his/her assessment of six adults who had been on ART for at least 6 months. This approach provides results for a best-case scenario. Sampling less experienced clinicians was expected to result in lower performance. We excluded children as paediatric treatment guidelines differ substantially from those for adults (Ministry of Health, 2011a).

HFs in both sub-regions were randomly selected without replacement. We sampled six patients per facility on a first come, first served basis assuming patients arrived randomly at HF; also our assessments took place at different times of the day. After informed consent, the same six patients per facility were interviewed, their reports checked and their clinical visits observed.

We pretested and refined the R-HFA tool May 13–14, 2015, followed by a 2-day training of the data collection team; all enumerators had a professional clinical background. Data collection took place from May 18 to June 17, 2015. All completed R-HFA tools were cross-checked by one of the co-authors for logical errors and inconsistencies and corrected with the enumerator when necessary; the data were then entered and cleaned from June 18 to July 10, 2015. For the LQAS classification analysis, we used Microsoft Excel 2010, and SPSS V22 for all other analyses.

This study was reviewed and approved by the authors’ institutes.

## Results

Of the HF sampled in Acholi (*n* = 11) two were hospitals, six Health Centres (HC)-IV and three HC-IIIs; in Lango (*n* = 10) three were hospitals, four HC-IVs and three HC-IIIs. Our sample included both high- and low-volume HF with patient numbers ranging from 15 to 400 per day. The mean consultation duration in both Acholi and Lango was 9 min (range: Acholi 3–22 min, SD = 4.3; Lango 2–28 min, SD = 5.2; median = 8 min in both sub-regions). Three outliers (one in Acholi: 52 min; two in Lango: 34 and 48 min) were excluded because the patients waited for laboratory results before being discharged and the clinician attended other patients in the meanwhile.

Compliance with the national guidelines was low in both sub-regions. For only 43.2% of the 37 guideline indicators measured did Acholi reach the 80% standard of performance. Lango was classified adequate for only 37.8% of the indicators. The guidelines’ indicators are categorized as Input, Performance, Outcome and Patient Management. The pattern of compliance with the guidelines shows little difference across the two sub-regions (Table 2).

**Table 2:** Health facility classifications for input, performance, outcome and patient management indicators or antiretroviral therapy provision

No.	Indicator label	Tool	Acholi (*n* = 11, *d* = 8)	Lango (*n* = 10, *d* = 7)
**Input**				
1a	Pre-service training	HWI	2 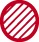	5 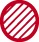
1b	In-service training	HWI	0 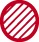	0 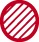
2	Supervision	HWI	1 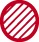	2 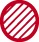
3	Laboratory supply (on site or referral system in place)	HFC	10 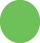	8 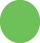
4	Equipment	HFC	6 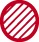	4 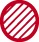
5ARV1	ARV availability first line (all essential combinations available)	HFC	6 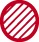	7 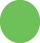
5ARV2	ARV availability second line (all essential combinations available)	HFC	7 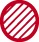	10 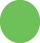
5TB	TB drug availability (all essential drugs available)	HFC	4 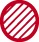	2 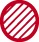
5OPP	Anti-opportunistic drug availability (all essential drugs available)	HFC	4 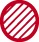	3 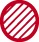
5FP	Family planning availability	HFC	5 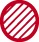	6 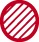
6ARV	Access to ARV (prescribed drugs available)	PI	11 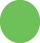	10 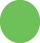
6OPP	Access to anti-opportunistic infection drugs (prescribed drugs available)	PI	11 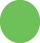	10 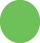
6Oth	Access to other medication (prescribed drugs available)	PI	9 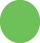	5 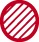
**Performance**				
7a	History taking: current well-being	Obs	11 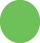	9 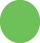
7b	History taking: hospitalization, changes	Obs	4 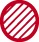	4 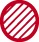
7c	History taking: TB screen	Obs	5 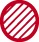	7 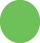
7d	History taking: symptom checklist	Obs	0 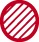	0 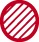
8	Physical examination	Obs	0 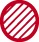	0 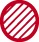
9a	Treatment: consistent with HW diagnosis	Obs	9 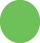	5 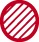
9b	Treatment: consistent with history and examination	Obs	3 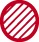	1 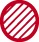
10a	Counselling: adherence strategies	Obs	7 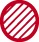	6 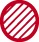
10b	Counselling: transmission of HIV	Obs	3 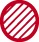	0 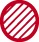
10c	Counselling: family testing	Obs	2 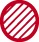	0 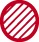
10d	Counselling: medication use	Obs	8 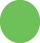	5 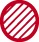
**Outcome**				
11a	Patient perception: history	PI	10 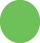	9 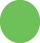
11b	Patient perception: examination	PI	6 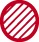	4 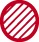
12	Patient knowledge: medication	PI	11 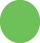	10 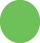
13	Patient’s adherence to medication	PI	10 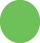	8 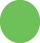
14	Disclosure of the status to partner	PI	11 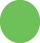	9 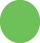
15	Family testing for HIV	PI	10 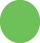	6 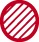
16	Patient knowledge: HIV	PI	0 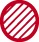	0 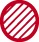
**Patient management**				
17	CD4-monitoring (last CD4-count not older than 9 months)	PR	2 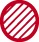	1 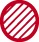
18	ART Initiation	PR	8 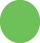	5 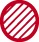
19a	ART documentation: TB screen	PR	11 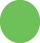	9 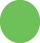
19b	ART documentation: treatment regimen	PR	11 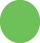	10 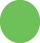
19c	ART documentation: counselling	PR	2 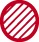	0 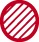
20	Follow-up	PR	9 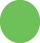	8 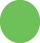

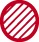
 = failure of a sub-region for that indicator, 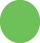 = success of a sub-region for that indicator. The sub-regions were appraised according to the upper and lower threshold (pU = 80%, pL = 50%, Acholi: *N* = 20, *n* = 11, *d* = 8; Lango: *N* = 21, *n* = 10, *d* = 7).

HWI, health worker interview; PI, patient interview; Obs, observation of the clinical visit; PR, patients’ records.

### Inputs

In addition to the shortage of HW, their training and supervision was problematic: Only two of the sampled facilities in Acholi and five in Lango had staff with accredited pre-service training for treating PLHA which is below the cut-off value of *d* = 8. In-service training did not take place in any sampled facility nor did regular supervision; only one HF in Acholi and two in Lango reported supervision meetings. However, all HW could indicate a contact person to discuss or refer difficult cases, which indicates functional management support.

We detected problems with the supply chain in Acholi. The essential first and second line ARV combinations (according to the national ARV guidelines) are: (first line) TDF/3TC, AZT/3TC, ABC/3TC, NVP, EFV, and (second line) ATV/r, LPV/r. Acholi failed for these provisions as the only consistently available drug combination was AZT/3TC. Lango did not exhibit this problem. However, patients in both sub-regions had access to their prescribed ARV combination which was AZT/3TC. Whether or not this treatment was the appropriate medication for the patient based on his/her clinical and immunological status stays unclear as neither the clinical nor the immunological monitoring took place.

Both sub-regions failed to reach the standard for the availability of the essential anti-opportunistic drugs. Nystatin and Dapson were available in <50% of the assessed facilities in Acholi, and Lango was below the threshold in Nystatin availability. Cotrimoxazole was the sole anti-opportunistic drug in adequate supply and the only one prescribed by HW in both regions.

As PLHA need to be monitored and managed for any (infectious) disease more closely than other patients, their access to drugs besides ARVs is also crucial. When assessing access to other drugs, Lango failed mainly due to the lack of analgesic drugs, but also for different anti-infective medications (e.g. antifungals, anthelmintic and antibiotic drugs). Acholi was not deficient in the latter medications.

Laboratories satisfied the basic requirements for monitoring PLHA (referral system or equipment complete). However, the availability of equipment needed to clinically monitor patients on ART (e.g. stethoscope, sphygmomanometer and the like) was inadequate in both sub-regions and the use of the laboratory or referral system for patient monitoring was near to zero (see below and Table 2).

### Performance

While clinicians dispensed to patients ARVs and other drugs, a complete patient history (e.g. recording symptoms/danger signs as suggested by the national ART guidelines) and a physical examination were not taken in any of the assessed HF. Even patients with complaints were not examined in most cases. Patients without explicitly stated problems were not checked at all. An attempt to detect (asymptomatic) danger signs on an early and regular basis did not take place. Table 2 shows four categories of essential history taking as per the ART guidelines and the results for each sub-region.

Furthermore, the treatment of symptomatic patients was problematic. Diagnoses were taken without sufficient patient history or physical examination in either sub-region. In addition, HW in Lango deviated from their noted diagnosis when prescribing medicine. This result raises the possibility of the improper use of medications. Also, in regards to the patient counselling, both sub-regions failed (Table 2).

### Outcome

Patients’ knowledge about HIV transmission was inadequate in all facilities. Ugandan guidelines require counselling all HIV patients on six ways to avoid the transmission of HIV: abstinence, faithfulness, condom use, consequent treatment with ART, use of ART to prevent transmission of HIV from mother to child during delivery and no sharing of sharps.

An average patient in Acholi could recall 2.6 of 6 HIV transmission pathways, and 2.3 in Lango. The predominant area of knowledge about HIV prevention in both regions was condom use. ‘No sharing of sharps’ was known in Acholi.

All patients knew correctly when and how to take the prescribed drugs. In both sub-regions, an adequate number of patients indicated adherence to treatment and disclosure of their status to their partners. In contrast to Acholi, Lango failed for HIV testing for all or most of the family members. Even though history taking was done poorly, patients felt sufficiently comfortable with it in each sub-region. However, patients’ in both sub-regions also felt their physical examination was inadequate; this finding is consistent with our own conclusion based on direct observation.

### Patient management

Even though 7 of the 11 assessed HF in Acholi and 6 out of 10 in Lango had functioning laboratory equipment and relevant reagents for a CD4-count available on the day of visit, almost none of them actually did so. HF without an on-site testing capability did not monitor patients’ CD4-counts at all, despite having a functioning referral system in place. Therefore, even though the indicator for laboratory supply (meaning on-site measurement or referral system in place) met the target, both sub-regions failed to monitor CD4-counts.

Both regions did achieve the standard for clinical TB screening when using the information available on patient records. However, our direct observation failed to confirm adequate clinical TB screening in Acholi; nevertheless, Lango continued to meet the 80% target. Both sub-regions failed for almost all aspects of counselling (i.e. adherence strategies, transmission of HIV, family testing) with the exception of counselling about drug administration in Acholi, which met the target. Also, both sub-regions failed for documentation of counselling (Table 2).

## Discussion

This cross-sectional health facility assessment survey in two northern Ugandan sub-regions evaluated the QoC for non-naïve ART patients and showed poor results in all indicator categories. These results suggest that Acholi and Lango are sub-regions needing additional support to improve the health systems leading to effective ART care. Other regions where we carried out the pre-test of the R-HFA, reported similar challenges (Deurman, 2011). Additional comparable assessments are now needed in Uganda to identify other geographical and technical areas in need of priority support. These results also suggest the need for QoC assessments elsewhere in sub-Saharan Africa.

Our assessment revealed discrepancies between the availability of the printed documentation and direct observation of performance. Observation of counselling in Acholi exhibited better performance, than the availability of documentation of counselling guidelines which failed to reach the national quality standard. However, while the documentation of TB screening reached the standard in both sub-regions, direct observation data indicated that Acholi failed to meet the standard for clinical performance. This discrepancy is in accordance with other studies (Edwards *et al.*, 2014). Appraisals using direct observation for child health care identified poor clinical performance, and noted discrepancies between reported and observed health care (Oladele *et al.*, 2012; Berendes *et al.*, 2014). Although the availability of quality documentation of clinical performance is widely seen as an indicator of good QoC, the use documentation can be hampered by high workload and/or complicated documentation regulations (Shihundla *et al.*, 2016). The QoC described and evaluated by examining documentation only, might lead to false-positive and/or false-negative results.

Similarly, the availability of services did not ensure their use. Regular immunological monitoring failed in both sub-regions, even though functioning equipment for monitoring was available in twice as many HF as those which failed; the other HFs reported a referral system in place although there is no evidence it was used sufficiently. More than 80% of HF could have carried out immunological monitoring (on-site or by referral) but did not do so. Hence, health system managers should not infer that the availability of a service leads to its correct and regular use. Data about available services at the HF level are not sufficient for making conclusions about QoC.

Another discrepancy we detected was the availability of ARVs and essential drugs to treat opportunistic infections. Acholi exhibited insufficient availability of ARVs in their supplies and stocked only one ARV combination sufficiently. Lango did not have this problem. However, clinicians in both sub-regions dispense the ARVs they had on hand without the benefit of giving a physical examination or monitoring immunological status.

A similar pattern was observed for the availability of essential anti-opportunistic infection drugs and other medications in Acholi. Both sub-regions failed for having the essential drugs as required by the national ARV guidelines, and again clinicians tended to dispense drugs that were available such as Cotrimoxazole (see Table 2). This might be a sign of a long-term supply chain problem, or HW could have grown accustomed to the absence of drugs and therefore tended to offer patients the available medication rather than the most appropriate therapy. This practice is described by other studies (Minior *et al.*, 2017). We can report that only three institutions in Acholi and one in Lango treated patients in accordance with clinical findings. As the appraisal of individual symptoms and the resulting treatment were not the main interests of our assessment, the study design does not allow us to make generalizations about this practice.

Clinical observation at a regional or nationwide level can be expensive and time consuming. Most QoC assessments, therefore, rely on input indicators and documentation only. However, the failure in using available technology and the discrepancies we detected leave questions about whether QoC can be sufficiently evaluated by counting the availability of documentation and service availability only. The method we used is field friendly, and time and HR efficient, and not costly. We assessed up to two HF in one working day. This is the same time expenditure for a team using WHO’s SARA tool (World Health Organisation, 2013) which does not include clinical observation which proved to be very insightful. Other HFA that include interaction with providers and patients and/or direct observation (HRHS, HFA) do consume more time per team (1–2 days per facility) (Hozumi *et al.*, 2006).

We examined QoC in reference to the national ART guidelines; as a result, the international standards were not included in the tool. This deficiency may limit the comparisons of this study with other settings. We also recognize that direct observation of clinical care may have produced a Hawthorne effect, although other studies of this effect have shown no to little impact of direct observation on improving results (McCambridge *et al.*, 2014; Goodwin *et al.*, 2017). Nevertheless, in our study should it exist, the Hawthorne effect may be an advantage. If our results record the best-case scenario then clinical performance without direct observation could be even worse.

The outcome indicators we used did not reflect the deficiencies in inputs and processes; patients were sufficiently content with history taking, knew when and how to take their drugs, and stated they took their medication. Reports from north-western Uganda (Ahoua *et al.*, 2009) revealed 8% of treatment failure after 1-year on ART and 38% after 2-years on ART; among the treatment failures, 7% (after 1-year) and 13% (after 2-years) showed a resistant virus. Extrapolating those findings to our study clientele suggests that our outcome indicators are incomplete, and underlines the priority of regular immunological monitoring.

The discrepancies between the reported and the observed QoC were surprising. The comparison was between reaching the 80% cut-off for one indicator and failing the cut-off for the counterpart observing indicator (or vice versa). Such results suggest that more in-depth mixed methods approaches might be needed to provide additional insight into the supply chain and prescribing practices we have noted.

In the light of the overall poor-quality health care for ART patients and the detected discrepancies, we recommend to further QoC studies of HIV services that include direct observation and immunological monitoring. The extent and impact of the deficiencies in quality on the patients’ status and outcomes need to be assessed and addressed. More regions should be involved in the sampling frame to see whether the northern Ugandan sub-regions are priority areas for technical assistance in HIV care or whether the QoC deficiencies is a wider spread problem throughout Uganda. Such implementation research should also take place elsewhere in sub-Saharan Africa, as well as accompanying studies of ARV resistance.

*Ethical approval.* This study was reviewed and approved by the Liverpool School of Tropical Medicine Ethical Board, The Makerere University School of Public Health Higher Degrees Research and Ethics Committee and the Ugandan National Council of Science and Technology.

## Supplementary Material

czz074_Supplementary_Data
